# Editorial: Recent advances on the multimodal search for markers of treatment response in affective disorders: from bench to bedside, volume II

**DOI:** 10.3389/fpsyt.2025.1597565

**Published:** 2025-08-28

**Authors:** Martin Walter, Sebastian Olbrich, Nils Opel, Maria Strauss, Bin Zhang, Lena Vera Danyeli

**Affiliations:** ^1^ Department of Psychiatry, University Hospital Jena, Jena, Germany; ^2^ Department of Psychiatry, Psychiatric University Hospital Zurich, Zürich, Switzerland; ^3^ Department of Psychiatry, University Hospital Leipzig, Leipzig, Saxony, Germany; ^4^ Tianjin Anding Hospital, Tianjin, China

**Keywords:** depression, biomarker, subtypes of depression, treatment resistance, imaging

Affective disorders and their comorbidities concern the largest group of psychiatric patients due to 1) the early and continuous lifelong high incidence of the disorders and 2) their chronic nature, which is often due to inadequate or delayed treatment responses (1–3). This contrasts with the comparably high level of treatment success during controlled treatment escalation, with response rates of 60-80% to ECT even in difficult-to-treat patients. Although the application of stratified treatment regimens—starting with relatively well-tolerated drugs or psycho(mono-)therapy—has been shown to be more effective compared to free treatment regimens (4), the majority of algorithms still begin with first-line drug suggestions that offer only moderate expected response rates. For these first-line treatments, remission rates are as low as 33% (STAR-D), decreasing further at subsequent treatment levels. Consequently, poor or significantly delayed remission rates add to a complex situation that recently resulted in as few as 7% of patients (across several diagnoses) achieving timely and efficient treatment on an international level (5). Not surprisingly, the lengthy search for the right individual treatment, alongside the side effects accompanying ineffective treatments, which may even worsen clinical status, leads to a higher number of patients who, disappointed, drop out of treatment courses that might eventually have led to a significant response. The search for (bio-)markers predicting individual treatment responses has spanned the careers of many clinicians and scientists and has led to some candidate markers, that, however, have relatively low predictive values or replicability (6). While factors predicting general treatment resistance have been reported, these, however, seem to have little value in supporting a shift towards more targeted interventions.

Given the fact that there is a very large group of patients who would, in principle, respond or remit once efficient treatments are identified and applied, and given the long course of ineffective treatment, during which patients lose adherence, identifying and implementing new, additional predictors promises substantial clinical and health economic benefits. These benefits are maximized when these markers not only allow early transition to escalated treatments but also identify subsystems that characterize biological patient subgroups with specific therapeutic targets that differ from those addressed by first- or second-line treatments. Earlier collections of such candidates have particularly demonstrated potential ways to incorporate additional knowledge into treatment prediction (7).

Over the past five years, evidence has emerged regarding 1) new mechanisms and candidates and 2) new treatment opportunities made possible by large-scale reimbursement options e.g. TMS or NMDA antagonists. These new opportunities and insights have fueled research into treatment rationales that go beyond monoaminergic approaches, identifying exciting new candidate markers related to glutamatergic modulation. New therapeutically driven routes towards glutamatergic mechanisms have emerged alongside growing insights into biological phenotypes underlying long-debated clinical subgroups of depression, mainly melancholic and atypical subtypes (8). Importantly, these subtypes, with distinct symptom and response profiles, have been increasingly characterized by multidimensional biological markers (9) that link regional brain imaging evidence, patient history and the molecular convergence of several biological systems. Ongoing evidence on patients who are best characterized as “immunometabolic” (10), suggests a convergence of a metabolic/adipose load with accompanying (low-grade) inflammatory signatures in these patients indicating the need for new and adapted treatment strategies. The emergence of neuropsychoimmunological patient characteristics has interestingly converged with the introduction of new treatments at the level of glutamatergic signaling, where activation of IDO is related to downstream changes in kynurenic metabolites with NMDA-ergic properties.

This development is reflected in the contributions to our second edition: Consequently, Reininghaus et al. expanded upon their previous characterization of kynurenic pathway abnormalities in depression (11) to explore their predictive potential for individualized treatment outcomes. They showed that statically altered kynurenic acid (KYN) can not only help identify non-responders, but that KYN increases over the course of treatment signifying treatment response.

Importantly, such metabolic signatures have recently become a focus of non-invasive imaging studies (12). In this Research Topic, Moreau et al. reviewed evidence from 20 different studies on imaging treatment predictors in OCD. As a result, they concluded that structural assessments failed to provide consistent markers of treatment response. Their findings suggest that relevant processes may be more effectively captured by dynamic i.e. metabolic or neurophysiological investigations. Such metabolic investigations have become possible through the introduction of feasible MR spectroscopy sequences (MRS), which can capture a wide range of metabolic markers in certain brain regions. Watling et al. applied this technique to an emerging candidate of metabolic abnormality, namely glutathione (GSH), the brain’s most abundant antioxidant. While they failed to find differential GSH levels in a group of PTSD patients, they reported initial evidence of altered metalloproteinase (MMP)-9 and myeloperoxidase (MPO) levels, which are also related to disease progression.

Previous accounts of the application of quantitative EEG in treatment prediction (13) have been followed up by the final contribution to this second volume: Kim et al. demonstrated that a QEEG allowed for successful identification of depression patients with an accuracy of 92.31% and a 10-fold cross-validation loss of 0.13%. While these numbers are already considerably higher than those reported for MRI-based imaging signals (14), the true power of electrophysiological signals, however, might be found in the correct labeling of treatment responders for various treatment options in depression (15, 16).

In conclusion, the most recent advances concern both new targets and techniques with an expanding set of potential biomarkers across multiple modalities, which, however, may depict different aspects of similar mechanisms or patient characteristics. Therefore, the search for suitable treatment markers to guide specific interventions remains an open quest with high potential pains and gains.
